# Embryo manipulation via assisted reproductive technology and epigenetic asymmetry in mammalian early development

**DOI:** 10.1098/rstb.2012.0353

**Published:** 2013-01-05

**Authors:** Takashi Kohda, Fumitoshi Ishino

**Affiliations:** Department of Epigenetics, Medical Research Institute, Tokyo Medical and Dental University, 1-5-45 Yushima, Bunkyo-ku, Tokyo 113-8510, Japan

**Keywords:** assisted reproductive technology, intracytoplasmic sperm injection, epigenetic asymmetry, genomic imprinting, X chromosome inactivation, zygotic gene activation

## Abstract

The early stage of mammalian development from fertilization to implantation is a period when global and differential changes in the epigenetic landscape occur in paternally and maternally derived genomes, respectively. The sperm and egg DNA methylation profiles are very different from each other, and just after fertilization, only the paternally derived genome is subjected to genome-wide hydroxylation of 5-methylcytosine, resulting in an epigenetic asymmetry in parentally derived genomes. Although most of these differences are not present by the blastocyst stage, presumably due to passive demethylation, the maintenance of genomic imprinting memory and X chromosome inactivation in this stage are of critical importance for post-implantation development. Zygotic gene activation from paternally or maternally derived genomes also starts around the two-cell stage, presumably in a different manner in each of them. It is during this period that embryo manipulation, including assisted reproductive technology, is normally performed; so it is critically important to determine whether embryo manipulation procedures increase developmental risks by disturbing subsequent gene expression during the embryonic and/or neonatal development stages. In this review, we discuss the effects of various embryo manipulation procedures applied at the fertilization stage in relation to the epigenetic asymmetry in pre-implantation development. In particular, we focus on the effects of intracytoplasmic sperm injection that can result in long-lasting transcriptome disturbances, at least in mice.

## 1. Introduction

The intracytoplasmic sperm injection (ICSI) technique was originally developed to investigate fertilization in various animals [1]. The first mammalian ICSI experiment was conducted in hamsters by Uehara and Yanagimachi in 1976 [2]. This technology has been successfully applied to the field of laboratory animal science and livestock breeding. Since the initial application of ICSI to humans in 1992 [3], this technique has become increasingly popular as the fertilization method of choice in assisted reproductive technology (ART), despite persistent concerns of an effect on development. The effects of ART are thought to be attributable to two causes. One is impact of the technique used, and the other is the underlying maternal or paternal subfertility [4].

Cohort studies of children conceived by ART have been conducted in an attempt to investigate the risks of genetic and epigenetic impairment. Recently, there was a report of a large ongoing cohort study in Australia on the effects of ART [5]. It is reported that the association between *in vitro* fertilization (IVF) and the risk of any birth defects was not significant after adjustment for confounding factors. However, the increased risk of ICSI-associated defects remained significant. This report is consistent with a previous cohort study [6] as well as the findings from a mouse model [7]. In addition, there is some evidence of an increased risk of imprinting disorders in ART children [4]. It is also reported that the birth-weight of singletons born after the transfer of frozen blastocysts was significantly higher when compared with singletons born after the transfer of fresh blastocysts [8]. However, it is not easy to draw conclusions about the impact of the individual components of the ART technique applied using merely epidemiologic studies.

It has been demonstrated that *in vitro* culture of the embryo has long-term effects in mice [9–12] and cows [13]. Gene expression alterations also are reportedly observed in IVF-conceived mice [14]. The methylation aberration of imprinted genes has also been reported in the case of embryo culture [15–17] and superovulation [18–21]. Recently, DNA methylation aberration in the imprinting control regions (ICRs) also has been observed in ICSI-conceived mice [22].

These data suggest that environmental factors in early mammalian development are crucial to epigenetic regulation, including but not only genomic imprinting. Thus, it is critically important to evaluate in detail the impact of ART on the genetic, epigenetic and phenotypic outcome in relation to genome-wide epigenetic regulation in early development. This is because it is the time period in which global changes in the epigenetic landscape occur in paternally and maternally derived genomes, and such epigenetic changes are potentially very sensitive to environmental factors. In this review, we describe the different epigenetic landscapes in the sperm and egg and between male and female pronuclei, which result in epigenetic asymmetry from the viewpoint of the DNA methylation and histone modification related to the mechanisms of genomic imprinting, X chromosome inactivation and zygotic gene activation (ZGA). Second, we summarize the effects of ICSI by comparing the effect of conventional IVF on epigenetic regulation in pre- and post-implantation development as well as in postnatal growth and behaviour. Finally, we discuss the primary ICSI effects on the regulation of gene expression related to ZGA.

## 2. The epigenetic landscape differs between the sperm and egg, and also between male and female pronuclei

**Figure 1.**
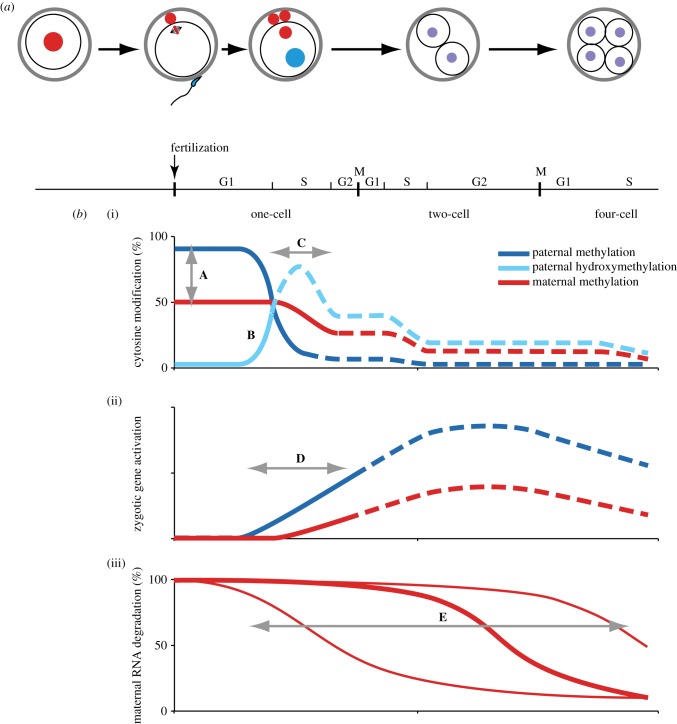
Schematic of DNA modification, gene expression and maternal RNA degradation in early mouse development. (*a*) A drawing that depicts the timing of fertilization, stage of development and the cell cycle. (*b*) (i) A schematic of the level of cytosine modification in early development. **A**, The mature sperm and oocyte have a different genome-wide cytosine methylation. **B**, Methyl cytosine of the paternally derived allele is rapidly converted into hydroxymethyl cytosine. **C**, The first DNA replication after fertilization take place in this period. The conversion of methyl cytosine to hydroxymethyl cytosine and DNA replication overlap. Cytosine in de novo synthesized DNA does not become modified and undergoes a ‘passive demethylation’ process in this phase, so the percentage of modified cytosine is reduced. (ii) Zygotic gene activation in the paternal and maternal alleles. **D**, In the zygote, the paternal pronucleus starts transcription earlier and is more active than the maternal pronucleus. At the two-cell stage, de novo mRNA is synthesized in a significantly large amount and with many more genes compared with the one-cell zygote. However, the detailed expression ratio of the paternal and maternal alleles at the two-cell stage or later is not yet reported. (iii) **E**, Degradation timing of most of the maternal mRNA. Some mRNAs degrade more rapidly and other mRNAs degrade more slowly (thin lines) than represented in the thick line.

The genome-wide DNA methylation process that takes place during germ cell maturation differs in sperm and oocytes [23]. Recently, detailed DNA methylation profiles were analysed using genome-wide bisulphite sequencing [24,25]. CpG islands (CGIs) are usually located in the promoter region and are hypomethylated in somatic cells. In general, the genome-wide DNA methylation of regions other than CpG islands, such as inter-genic regions, was shown to be higher in sperm (90%) than in oocytes (40%; figure 1*a*) [25]. Recently, Kobayashi *et al.* [25] reported that there are several sperm-specific and oocyte-specific methylated CGIs (sperm-specific = 818/23 021, oocyte-specific = 2014/23 021, both methylated = 377/23 021). The number of hypermethylated CpG islands in oocytes (approx. 10%) is relatively high compared with somatic cells such as fibroblasts (approx. 3%). The number of differentially methylated CGIs is much larger in the sperm and oocyte than in the previously reported methylated CGIs linked with genomic-imprinted regions. Some of these differentially methylated CGIs are not methylated at the pre-implantation stage. However, a significant number of oocyte- (817) and sperm-specific (34) methylated CGIs also persist in early development, such as in the ICRs [25]. The roles of both these stable and unstable differentially methylated CGIs in non-imprinted loci for the regulation of pre-implantation gene expression are presently unknown. It is known that gene expression and gene-body DNA methylation are in good correlation in somatic cells. In the oocyte, there was a close relationship between gene-body DNA methylation and gene expression, while the correlation of gene-body methylation and gene expression was poor in sperm, probably due to the genome-wide hypermethylation [25].

**Figure 2.**
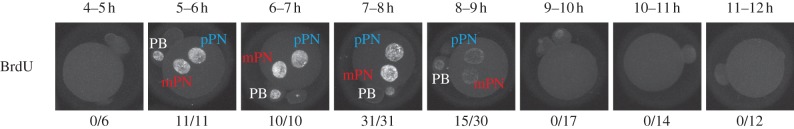
DNA replication timing in the one-cell zygote. A zygote was pulse-labelled by bromodeoxyuridine (BrdU) for 1 h, immediately fixed by formaldehyde and stained with an anti-BrdU antibody. The pulse-label time intervals from the moment of fertilization are indicated over the pictures. The numbers below the pictures represent ‘BrdU positive zygote’/‘zygote examined’. This result shows that DNA replication starts at 5–6 h after fertilization in both the paternal and maternal pronuclei and terminates at 8–9 h after fertilization. pPN, paternal pronucleus; mPN, maternal pronucleus; PB, polar body.

At the one-cell stage, 5-methylcytosine (5mC) in the male pronucleus is rapidly converted to 5-hydroxymethylcytosine (5hmC; figure 1*b*), and this conversion is not observed in the female pronucleus. The Tet1, Tet2 and Tet3 enzymes catalyse the conversion reaction from 5mC to 5hmC. Experimental investigation using both an anti-5hmC antibody and an anti-5mC antibody clearly demonstrated that the rapid disappearance of the 5mC in the male pronucleus is due to the conversion to 5hmC by the Tet3 enzyme [26,27]. Maternal germ-cell-specific *Tet3* disruption results in there being no preferential 5mC conversion to 5hmC in the paternal pronucleus. Oocytes lacking the Tet3 enzyme exhibit a severe effect on embryonic development [28]. The conversion reaction in the paternal pronucleus starts at 4–6 h after fertilization, and a marked asymmetry in the male and female pronucleus is clearly evident 8 h after fertilization. The first DNA replication also starts in almost the same period in both pronuclei [29,30]. In an unpublished experimental result, the DNA synthesis started at 5–6 h after fertilization and terminated before 9 h (figure 2; T. Kohda 2012, unpublished data). An ‘active demethylation’ mechanism was reportedly proposed in this process, in which 5mC is converted to thymine by activation-induced cytidine deaminase and then the TG mismatched base pair is restored by a DNA repair mechanism [31]. It is also reported that 5hmC is further oxidized to 5-formylcytosine and 5-carboxylcytosine in the paternal allele by Tet enzymes [32,33]. However, several lines of evidence have suggested that most of the 5hmC in the male pronucleus as well as 5mC in the female pronucleus is subjected to ‘passive demethylation’ [26,27,34] (figure 1*c*).

This asymmetric change from 5mC to 5hmC was observed in many mammalian species, including humans, mice and rats, but not in sheep or rabbits [35–37]. It also was induced in the ICSI zygote in mice and rats, although its efficiency is relatively low in the latter [38–41], but it does not occur in zygotes fertilized by round spermatid injection (ROSI) in mice [39,42]. The efficiency of full-term development of ROSI is significantly lower than that of ICSI, and this may be partly explained by the loss of the conversion reaction in the paternal pronucleus [28].

The rapid conversion to 5hmC was also observed in the transferred donor nucleus in somatic-cell cloning by nuclear transfer. In this case, both of the two alleles of the donor nucleus were subjected to hydroxylation, with the result that there was no epigenetic asymmetry except in the imprinted regions [43,44]. This rapid conversion was also catalysed by the Tet3 enzyme [28]. The fact that most somatic clone embryos exhibit lethality at the implantation stage due to abnormal X chromosome inactivation and that *Xist* knockout and knockdown significantly improve the efficiency of somatic cloning are also suggestive of the importance of the epigenetic asymmetry between the two parental genomes for proper *Xist* gene expression and resulting X chromosome inactivation in pre- and post-implantation development [45,46].

In addition to cytosine modification, histone modification also results in an asymmetry between paternal and maternal alleles in early development. In sperm, the genomic DNA is tightly packaged with protamines rather than with histones. Just after fertilization, the tightly packaged paternal chromatin delivered by the sperm is decondensed, and global protamine-to-histone exchange takes place. The paternal chromatin incorporates hypomethylated histones and has a low level of H3K9me2/3. During this period, the maternal chromatin remains relatively stable and has a higher H3K9me2/3 level compared with the paternal pronucleus [47]. *Dppa3* (*Stella/PGC7*) may play an essential role in protecting maternal alleles from the hydroxylation of mC in early development [48]. Recently, Nakamura *et al.* [49] demonstrated that Dppa3 protein binds to the maternal pronucleus chromatin via the H3K9me2. It is known that a small amount of histone remains in specific loci of the sperm nucleosome, including the imprinted regions [50,51]. These histones are able to retain their modifications and thus transmit ‘epigenetic memory’ via sperm, and as a result may contribute to the epigenetic asymmetry of the early embryo.

## 3. Genomic imprinting and X chromosome inactivation during early embryonic stage

Genomic imprinting and X chromosome inactivation are typical examples of parental asymmetry in the epigenome, the mechanisms for which are unique to mammals. In genomic imprinting, functional complementation between paternal and maternal epigenotypes is required for normal development, growth and behaviour, because certain paternally and/or maternally expressed genes (*PEGs* and *MEGs*) play essential roles in these processes, as discussed elsewhere in this issue [52,53]. Genomic imprinting is established in germ cells before fertilization, mainly as a difference in DNA methylation between the sperm and oocyte genome. In X chromosome inactivation, suppression of one X chromosome of the two female X chromosomes is required for gene dosage compensation between the male (XY) and female (XX) cells. This is also essential for cellular differentiation in both embryonal and placental cell lineages: only pluripotent cells, such as embryonic stem (ES) cells, induced pluripotent stem (iPS) cells and primordial germ cells (PGCs) have two active X chromosomes [54,55]. Non-mammalian organisms adopt other gene dosage compensation mechanisms, such as downregulation of each of the two female X chromosomes in *Caenorhabditis elegans* and upregulation of the one male X chromosome in *Drosophila melanogaster*.

Parthenogenetic and androgenetic embryos have only maternally and paternally derived genomes, respectively, but they can develop into blastocysts. ES cells can also be established from these embryos. Thus, it seems that the different functions in the paternal and maternal genomes might not be strictly necessary for pre-implantation development. However, soon after implantation, these uniparental embryos died, with severe but different morphological abnormalities in the placenta and embryo [56–58]. Furthermore, the cloned mouse embryos by nuclear transfer using day 12.5 PGCs, that have no parental imprinting memories, also died at post-implantation stage [59]. At this stage, parental imprinting patterns in PGCs are completely absent, exhibiting a unique gene expression profile in the default state of genomic imprinting that is different from that of either parthenogenetic or androgenetic embryos [59,60]. This evidence demonstrates that genomic imprinting is indispensable for post-implantation development.

As mentioned earlier, there are a large number of genomic regions that are differentially methylated in the sperm and oocyte in addition to the imprinted loci, but the imprinting markings in the zygote are not removed during the developmental process and throughout the lifetime. Targeted disruption of *Dppa3* resulted in the loss of imprinting in some imprinted genes, demonstrating that Dpp3 is essential for maintenance of imprinting memory during early development [53,61].

In mice, the paternal X chromosome is selectively repressed during early embryonic development and in placental lineage cells, whereas random X chromosome inactivation occurs in the embryonic lineages. X chromosome inactivation mainly depends on the allelic expression of the non-coding RNA named *Xist* [62–64], and the paternally derived *Xist* gene is preferentially expressed in the pre-implantation stage female embryo [65,66]. However, imprinting patterns around the *Xist* gene, such as a differentially methylated region, have not been elucidated, indicating that there is a DNA methylation-independent mechanism of the imprinted expression of *Xist* [67]. Subsequently, both the *Xist* alleles are repressed, and the two X chromosomes are transiently activated in the inner cell mass (ICM) of the blastocyst. The re-establishment of X chromosome inactivation in somatic cells then occurs randomly.

How are the two parental X chromosomes distinguished in female zygotes and embryos? In male germ cells, two sex chromosomes, X and Y, are inactivated by a *Xist*-independent mechanism known as meiotic sex chromosome inactivation (MSCI) [68,69]. MSCI occurs at the meiotic pachytene stage, when synaptonemal complexes bind all of the autosomes in a pairwise manner. The X and Y chromosomes have only a few homologous regions, called pseudoautosomal regions, and are regionally separated from the other autosomes so as to form a transcriptionally inactive XY body [70]. After meiosis, the sex chromosome in the male germ cell is repressed in postmeiotic sex chromatin until the elongation of the spermatid [71]. There is no MSCI in female germ cells, because the two X chromosomes form synaptonemal complexes such as the other autosomes. Therefore, it is highly probable that paternally and maternally derived X chromosomes have different epigenetic marks, and thus behave differently in zygotes. It is also reported that X chromosome inactivation at the pre-implanted stage proceeds in two steps, one of which is *Xist*-independent and the other is *Xist*-dependent [72]; the former is suggested to be originated in the MSCI process. In both cases, the genes on the X chromosome in female pre-implantation embryo display a paternal–maternal asymmetry, and genes on the X chromosome behave as maternally expressed genes [65,66].

Recently, there have been reports indicating that imprinted X chromosome inactivation exists even in somatic-cell lineage(s), suggesting that the imprinted patterns for X chromosome inactivation remain after ICM differentiation. For example, it is reported that X inactivation is skewed to the paternally derived X chromosome in the brain [73,74] and mammary epithelia [75], and thus the epigenetic X chromosome asymmetry may play a role even in adult tissues.

Although the effects of aberrant X chromosome inactivation are not apparent in the pre-implantation stage, X inactivation is also essential for post-implantation development in females, as is genomic imprinting. It is probable that the existence of two epigenetically different X chromosomes is necessary for the proper control of *Xist* expression in pre-implantation embryos, because aberrant *Xist* expression is observed in both parthenogenetic and androgenetic embryos [76,77]. This might be related to the fact that ectopic expression of *Xist* in the pre-implantation-cloned embryo is the major cause of the low somatic-cell-cloning efficiency mentioned earlier [45,46].

Aberrations of the monoallelic expression of imprinted genes induce distinct congenital diseases. There is increasing evidence of a link between ART and imprinting diseases, such as Beckwith–Wiedemann syndrome [78–88], Angelman syndrome [83,89,90] and Silver–Russel syndrome [91–93]. On the other hand, other studies have reported that there is no increased incidence of ART-related imprinting disorders after correction of the fertility problems of the parents [94]. Recently, it was reported that there was a DNA methylation aberration in the ICRs induced by ICSI in mice [22]. The degree of the effect is low, but the incidence of the aberration is relatively high compared with imprinting disorder observed in human ART. It is possible that a low degree of DNA methylation aberration without any apparent phenotypic disorder may also occur in human ART. Well-designed DNA methylation analyses in human ART are needed. There have been no reports so far that ART affects X chromosome inactivation.

## 4. Long-lasting intracytoplasmic sperm injection effects on gene expression regulation

An epidemiological approach, such as has been used in many of the cohort studies carried out on children conceived by ICSI and/or IVF, is limited in distinguishing the causes of the observed effects, because it is difficult to determine whether they are really caused by the technology itself or originate from either genetic abnormalities or risk factors intrinsic to the patients. Obviously, other factors, such as genetic background and the surrounding environment of the children, have an effect on the results of cohort studies. Therefore, it is necessary to investigate the possible influence of ART on development using a genetically homogeneous model system, such as mice, under strictly regulated conditions.

**Figure 3.**
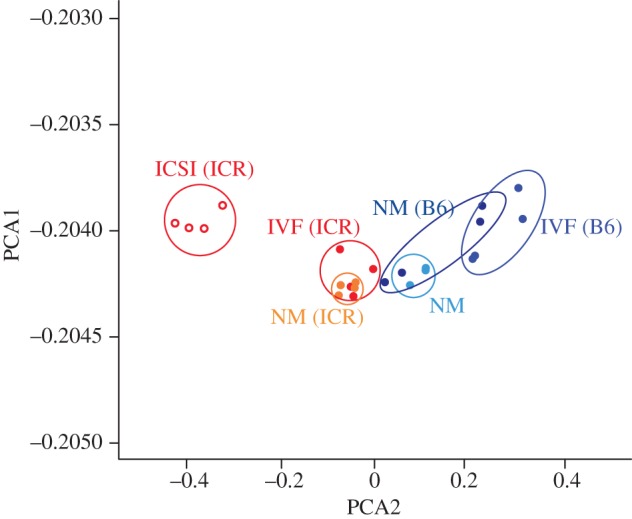
Principal component analysis of the gene expression profile. The transcriptional profiles are shown of the neonatal brain of mice conceived by ICSI, IVF or natural mating (NM) and embryo transfer to the surrogate mothers. Vertical axis (PCA1) and horizontal axis (PCA2) are principal component 1 and 2, respectively, of the gene expression profiles obtained by DNA microarray. Genetic background of all neonates are B6×D2. Strain name of surrogate mothers (B6 or ICR) are indicated in parentheses. NM: natural mating and continued the pregnancy. NM (B6): natural mating, flush out embryos and transplant to B6 surrogate mothers. NM (ICR); natural mating, flush out embryos and transplant to ICR surrogate mothers.

Conventional IVF and ICSI procedures are composed of several common artificial techniques, such as super ovulation, collection of the unfertilized eggs, *in vitro* culture and transfer to surrogate mothers, in addition to the process of fertilization itself. For proper assessment of the effects of IVF and ICSI, it should be taken into account that other experimental procedures are carried out under the same conditions, such as the overall *ex vivo* embryo-handling, including the *in vitro* culture conditions and transplantation to the surrogate mothers. The gene expression profiles should be compared between neonates from natural mating and those conceived by conventional IVF. In our analysis, a comparison was carried out using three neonatal organs, the brain, liver and kidney. In these experiments, fertilized eggs from natural mating were collected and transplanted to surrogate mothers in the same manner as those from IVF. Embryos were cultured for one day and transferred at the two-cell stage to minimize the effect of *in vitro* culture. As shown in figure 3, no difference was observed between the gene expression profiles of these two, indicating that the conventional IVF technique itself is a safe method, at least under the limited *in vitro* culture condition of a single day. The only differences we observed were dependent on the strains of the true or surrogate mothers.

However, the gene expression profiles of ICSI-conceived pups were significantly different compared with the pups conceived by natural mating and IVF. The gene repertoire affected by ICSI was different in each organ, and the genes exhibiting a more than twofold change was in the range of 3–5% in each organ. These gene expression changes induced by ICSI diminished by the eight-week stage in the B6 strain. In the case of B6D2F1, a certain effect persisted in the brain. The mechanism for this effect on the genes is not known, but there was a significant number of overlapping cases between the genes affected by ICSI and the genes showing large gene expression changes in this period. It is possible that the major part of the ICSI effect at the neonatal stage reflects the consequences of a gene expression cascade starting from a change initially induced in a small number of genes at the fertilization step. The gene expression changes induced by the ICSI procedure were not transmitted to the next generation by sexual reproduction to the extent examined. The ICSI effects on gene expression were observed as early as in the blastocyst stage. These observations indicate that an event at the time of fertilization can affect the genome and induce long-lasting changes in gene expression via an epigenetic mechanism or other distortion of the gene expression network.

It is important to assess whether ICSI induces not only an epigenetic and/or transcriptional difference but also phenotypic changes. We conducted comprehensive phenotypic analyses, including systematic behaviour evaluations comparing IVF and ICSI mice using the B6 strain. There were no significant phenotypical differences between the ICSI and IVF mice, except for a slight reduction of spontaneous activity in the home-cage. The behavioural analyses were performed simultaneously under strict conditions and a small but significant difference was observed. However, the difference induced by ICSI was within the range of the characteristics of this strain and all the other data indicated that there was no difference between ICSI and IVF, and thus the young adult ICSI mice were phenotypically essentially normal [7]. The genes affected by ICSI vary, depending on the mouse strains from which the sperm was isolated. This suggests that the ICSI effect on the epigenetic regulation of the sperm-derived paternal allele occurs at the fertilization step [7].

Fernández-Gonzalez *et al.* [95] investigated the long-term consequences of ART on gene expression as well as the behaviour of mice generated by ICSI using frozen–thawed sperm without a cryoprotector, as models of sperm with DNA damage. They observed a delay of 2 h on the ‘active demethylation’ of the male pronucleus in the embryos produced by ICSI. Moreover, ICSI affected both gene transcription and embryonic growth. The causes of the observed effects apparently included both the DNA fragmentation of the sperm and the ICSI procedure itself [95]. Giritharan *et al.* [96] also reported gene expression changes at the blastocyst stage induced by ICSI treatment in a different mouse, i.e. CF1 × C57BL/6. The total number of affected genes was similar to the result in our study, although different genes were up- or downregulated, presumably due to differences in the genetic background.

## 5. Zygotic gene activation at the two-cell stage and the effects of embryo manipulation

What is the cause of the ICSI effect? Allelic gene expression difference at ZGA as a result of epigenetic asymmetry may be present although there have been no direct reports. Then, is it possible that the ICSI effect directly and/or indirectly impacts on the ZGA leading to imbalanced gene expression for parental alleles? The ICSI procedure bypasses the acrosome reaction at fertilization and introduces certain sperm components into the egg cytoplasm. There are reports that sperm chromatin decondensation is slightly affected by the ICSI procedure in a strain-dependent manner [97,98]. As mentioned earlier, hydroxymethylation in the male pronucleus is affected by the ICSI in the rat [40]. The calcium oscillation is also altered by the ICSI procedure and may lead to changes in the gene expression profile [99]. This strain-specific alteration in the calcium oscillation induced by ICSI may account for our observation of strain-dependent transcriptome perturbation. It was observed that the ICSI-induced transcriptome perturbation also depends on the genetic background of the given mouse strain [7]. This suggests that the primary effect of the ICSI procedure on the regulation of gene expression may be biased to the paternal pronucleus in the zygote.

It had been reported that zygotic gene expression starts earlier in the male than the female pronucleus and that transcriptional activity is relatively higher in the male pronucleus [100] (figure 1*d*). A detailed genome-wide analysis of epigenetic modification and allelic expression has yet to be reported and is much needed, especially after the first cell division.

At the same time, a large amount of maternally stored RNA is present in the one-cell to two-cell stage embryo and is rapidly degraded at an early developmental stage (figure 1*e*). ZGA has been analysed by indirect methods using transcription inhibitors, because the zygotic mRNA from the maternal allele this stage is difficult to distinguish from the maternally stored RNA. As discussed earlier, epigenetic regulation of paternal- and maternal-derived alleles is quite different. To acquire an accurate view of the epigenetic landscape of the early embryo, it is critically important to separately analyse the paternal and maternal alleles during the ZGA period.

If ICSI exerts a different impact on the paternally and maternally derived allele, this should be testable by analysing allelic expression during the ZGA process, at least for the expression of the paternal alleles. For the effects on the maternal allele, changes in the total amount might be detectable, which would represent both the degradation of maternally stored RNA and the zygotic activation of the maternal allele. We recently set up the experimental system to see a paternal and maternal allele difference by means of transcriptome analysis with RNA-seq using single-nucleotide polymorphism (SNP) in a two-cell stage mice embryo comprising fertilized C57BL/6 oocyte with DBA2 sperm. Using this system, we have also obtained preliminary results indicating that ICSI differentially impacts the mRNA levels of paternal and maternal alleles. This observation suggests that the ICSI procedure at least exerts an effect on the ZGA of paternally derived genomes (T. Kohda 2012, unpublished data).

It is suggested that the zygotic expression of the genes of the paternal allele are delayed or reduced in the ICSI embryo. It is possible that the allelic asymmetry of the ICSI effect on the two-cell stage embryo is the result of a difference in epigenetic sensitivity between the male and female pronucleus at the one-cell stage or a reflection of an allelic difference in the B6 and D2 strains. The ICSI effects on the hydroxymethylation of the male pronucleus remain to be analysed in order to determine the mechanism of the ICSI effect on the regulation of gene expression. Approximately 90 per cent of the DNA methylation of the paternal and maternal alleles is erased by the blastocyst stage, and many early epigenetic asymmetries are simply absent, except in the case of imprinted genes. It is necessary to determine both the extent and which genes are affected in the long-term. As a considerable number of genes are affected by the ICSI procedure, the risk of phenotypic aberration will have to be taken into consideration. Genes that are sensitive to the gene dosage may show haploinsufficiency phenotypes. In the case of dosage-insensitive genes, monoallelic expression may cause an increased potential risk for loss of function. As an extreme example, if a tumour suppressor gene was to undergo such silencing, long-term risk for tumourigenicity could well be increased.

## 6. Future prospects

Many lines of accumulated evidence suggest that epigenetic effects are induced by ART procedures. There are many confounding factors, such as parental subfertility, age and crosstalk between epigenetic responses and genetic variations. All of these factors should be considered in investigations carried out to elucidate the effects of ICSI. Even considering these difficulties, however, recent progress in high-throughput sequencing is opening the way to an analysis of allelic expression and/or allelic epigenetic modification in the entire developmental process. This will eventually make it possible to assess the impact of the techniques used in ART on the epigenome and gene expression in humans.

As discussed in this review, the epigenetic and transcriptional asymmetries of paternal and maternal alleles may be important in early development. Such asymmetries, except for genomic imprinting, are thought to be for the most part diminished before implantation in normal development, but detailed allelic analysis may yet elucidate a novel parental asymmetry even in a later stage, such as mammary-gland-specific paternal X chromosome reactivation. Allele-specific expression (ASE) or differential allelic expression have been reported in the early embryo [101] and somatic cells or tissues [102,103]. It is possible that these kinds of biased expression are not unique but, rather, are also present in other tissues. However, caution is necessary, because allelic gene expression analysis using SNP tends to produce many false-positive ASE findings [104].

External factors affecting fertilization or early development, such as the ICSI procedure, may alter epigenetic regulation in such a manner that these effects persist in the course of development. Environmental stimuli such as ICSI may affect only one of two alleles and induce ASE under specific conditions. Thus, a comprehensive investigation of the multi-layered allelic asymmetries and their sensitivity to environmental factors in the early embryo will be necessary. Such a study would provide a novel view of the epigenetic landscape of the genome in mammalian development.
